# Effect of Ambient Temperature on the Thermoregulatory and Locomotor Stimulant Effects of 4-Methylmethcathinone in Wistar and Sprague-Dawley Rats

**DOI:** 10.1371/journal.pone.0044652

**Published:** 2012-08-31

**Authors:** M. Jerry Wright, Deepshikha Angrish, Shawn M. Aarde, Deborah J. Barlow, Matthew W. Buczynski, Kevin M. Creehan, Sophia A. Vandewater, Loren H. Parsons, Karen L. Houseknecht, Tobin J. Dickerson, Michael A. Taffe

**Affiliations:** 1 Committee on the Neurobiology of Addictive Disorders, The Scripps Research Institute, La Jolla, California, United States of America; 2 Department of Chemistry, The Scripps Research Institute, La Jolla, California, United States of America; 3 Department of Pharmaceutical Sciences, University of New England, Portland, Maine, United States of America; University of Chicago, United States of America

## Abstract

The drug 4-methylmethcathinone (4-MMC; aka, mephedrone, MMCAT, “plant food”, “bath salts”) is a recent addition to the list of popular recreational psychomotor-stimulant compounds. Relatively little information about this drug is available in the scientific literature, but popular media reports have driven recent drug control actions in the UK and several US States. Online user reports of subjective similarity to 3,4-methylenedioxymethamphetamine (MDMA, “Ecstasy”) prompted the current investigation of the thermoregulatory and locomotor effects of 4-MMC. Male Wistar and Sprague-Dawley rats were monitored after subcutaneous administration of 4-MMC (1–10 mg/kg ) using an implantable radiotelemetry system under conditions of low (23°C) and high (27°C) ambient temperature. A reliable reduction of body temperature was produced by 4-MMC in Wistar rats at 23°C or 27°C with only minimal effect in Sprague-Dawley rats. Increased locomotor activity was observed after 4-MMC administration in both strains with significantly more activity produced in the Sprague-Dawley strain. The 10 mg/kg s.c. dose evoked greater increase in extracellular serotonin, compared with dopamine, in the nucleus accumbens. Follow-up studies confirmed that the degree of locomotor stimulation produced by 10 mg/kg 4-MMC was nearly identical to that produced by 1 mg/kg *d*-methamphetamine in each strain. Furthermore, hypothermia produced by the serotonin 1_A/7_ receptor agonist 8-hydroxy-*N*,*N*-dipropyl-2-aminotetralin (8-OH-DPAT) was similar in each strain. These results show that the cathinone analog 4-MMC exhibits thermoregulatory and locomotor properties that are distinct from those established for methamphetamine or MDMA in prior work, despite recent evidence of neuropharmacological similarity with MDMA.

## Introduction

Discussions of the recreational drug **4-methylmethcathinone** (4-MMC; mephedrone, “miaow-miaow”, MMCAT, etc.) started to increase in online user forums such as bluelight.ru in 2008 [1]. Google Trends shows that searches for mephedrone accelerated sharply from mid 2009 through April of 2010 and news accounts spiked in the first quarter of 2010 as well [2]. This was due to increasing availability and use of the drug in the United Kingdom where it was legal to possess (marketed as “plant food”) until April of 2010. News or US Drug Enforcement Administration (DEA) accounts of the drug show 4-MMC has also appeared in the United States in Oregon [3], Central Texas [4] and North Dakota [5]. A press release issued by the United States Office of National Drug Control Policy noted that six states had taken action to ban 4-MMC as of February 2011 [6] and the DEA has requested information on several novel cathinone-derivative drugs of abuse [7].

As was emphasized by the initial reports from the UK Advisory Council on the Misuse of Drugs [8] and the Europol-European Monitoring Centre for Drugs and Drug Addiction [9], relatively little scientific information on any of the cathinone derivative compounds, and nothing specific for 4-MMC, was available until very recently [10]–[12]. An understanding of the effects of 4-MMC, at present, is therefore mostly by reference to the parent compound cathinone (found in khat, a plant material chewed for its stimulant properties in the Horn of Africa and Arabian peninsula) and the closely related analog, methcathinone. Cathinone is structurally similar to amphetamine and experience from the substituted amphetamine compounds suggests that modifications to the core structure can confer very different pharmacological properties, subjective experiences, abuse liabilities and toxicological risks. It is therefore of paramount importance to understand more about 4-MMC as well as additional cathinone derivatives (e.g., 3,4-methylenedioxypyrovalerone; MDPV, “bath salts”) that are starting to emerge or might be likely to emerge as popular recreational drugs.

There are indications that 4-MMC disrupts thermoregulation in some users, with both excessive heat (complaints about sweating) and cold reported [13]. About 15% of one experienced-user sample reported cold extremities while 67% reported excessive perspiration as a consequence of using mephedrone [14]. This mixed effect on thermoregulation is reminiscent of 3,4-methylenedioxymethamphetamine which can produce elevated or reduced body temperature in rats, depending on the ambient temperature and the dose administered [15], [16]; MDMA has also been shown to restrict blood flow to the periphery in rabbits [17] and rats [18]. Interestingly, some users report the subjective properties of mephedrone to be somewhat akin to those of MDMA [13], [19], [20]. This suggests the possibility that 4-MMC has properties similar to so-called empathogenic amphetamines whereas available data suggest that cathinone and methcathinone are more similar to classical amphetamine-type psychomotor stimulants [21], [22]. The user experiences add support to the hypothesis that 4-MMC may have pharmacological properties that are distinct from the classic amphetamine psychostimulants.

Recent studies *in vivo* and *in vitro* confirm the MDMA-like character of 4-MMC but also illustrate some differences. For example 4-MMC increases extracellular serotonin in rat nucleus accumbens with about the same efficacy as MDMA, but to a greater extent than serotonin is increased by amphetamine (AMP) or methamphetamine (METH) [11], [23]. Evidence on the effect of 4-MMC on extracellular dopamine is mixed since it increased dopamine about as much as did AMP but about 6-fold less than METH [11], [23]. It was also found in those studies that 4-MMC stimulated locomotor activity to approximately the same extent as did MDMA, in both cases much less than AMP/METH. *In vitro* studies showed that although 4-MMC exhibits greater relative *affinity* for the dopamine transporter relative to the serotonin transporter, it is more potent inhibitor of serotonin versus dopamine *uptake*; 4-MMC also exhibits a 15-fold greater affinity for 5-HT2 receptor subtypes than D2 receptors [12]. A repeated, high-dose binge exposure regimen under high ambient temperature resulted in hyperthermia, decreases in serotonin and SERT function in the hippocampus but did not alter dopamine content or DAT function in the striatum [10]. Together these initial findings are similar to prior reports for MDMA [24]–[26].

A study was conducted to determine the effects of 4-MMC on thermoregulation and locomotor activity in rats. Prior studies have shown that temperature responses to METH and MDMA depend on ambient temperature with hypothermia observed at or below normal laboratory temperatures of 22–24°C and hyperthermia above about 26°C in rats [15], [16], [27]; although this does not appear to be the case for monkeys or humans [28], [29]. The goal was to evaluate broader dose-response functions under two different ambient temperature ranges given that Kehr et al (2011) and Baumann et al (2012) examined only 0.3–3 mg/kg in locomotor assays and Hadlock et al (2011) examined the thermoregulatory effects of repeated high doses (10 or 25 mg/kg, 4X per day at 2 hr intervals) at a single (high) ambient temperature. Since the available neurochemical data are limited to a highest dose of 3 mg/kg 4-MMC, an investigation of the neurochemical effects of the highest dose used in the telemetry studies was included. Finally, additional drug challenges were included as positive controls to compare the locomotor effects with those of d-methamphetamine, a prototypical locomotor stimulant drug of abuse, and the thermoregulatory effects observed with 8-hydroxy-*N*,*N*-dipropyl-2-aminotetralin (8-OH-DPAT), a mixed serotonin 1A/7 receptor agonist which reliably induces hypothermia in rats. Experiments were conducted in two common strains of laboratory rat (Wistar, Sprague-Dawley) to determine the generality of the effects.

## Methods

### Animals

Male Wistar (N = 8; Charles River; New York) and Sprague Dawley (N = 8; Harlan; Livermore, California) were housed in a humidity and temperature-controlled (22°C±1) vivarium on a reverse light/dark cycle for the temperature and activity studies. Additional male Wistar Han (N = 3; Charles River; New York) and Sprague Dawley (N = 3; Charles River; New York) were housed in a humidity and temperature-controlled (22°C±1) vivarium on a regular light/dark cycle for the pharmacokinetic experiments. Rats were 9–10 weeks of age with an average bodyweight of about 300 grams at the start of the study. Animals had *ad libitum* access to food and water in the home cage.

### Ethics statement

All procedures were conducted under protocols approved by the Institutional Care and Use Committees of The Scripps Research Institute and University of New England and consistent with the *National Institutes of Health Guide for the Care and Use of Laboratory Animals* [30]. Once exception to the latter is that the ambient temperature manipulation diverged from the recommendations of the Guide but was explicitly approved under the IACUC protocol.

### Surgery

Sterile radiotelemetry transmitters (Data Sciences International CTA-F40) were implanted under general anesthesia (isoflurane 5% induction, 1–3% maintenance). The hair from the subxyphoid space to the pelvis was shaved and disinfected with povidone-iodine solution and alcohol and a sterile drape positioned. An incision was made along the abdominal midline immediately posterior to the xyphoid space, just large enough to allow passage of the miniature transmitter which was placed in the abdominal cavity. Absorbable sutures were used to close the abdominal muscle incision and skin was closed with non-absorbable suture. A minimum of 7 days was allowed for surgical recovery prior to starting the study.

### Procedure

For experiments, the ambient temperatures were established for a given condition by space heater and monitored with a temperature probe connected to the telemetry system. Each subject was transported to the experimental room for recordings made via placement of the telemetry receiver plate under a normal shoebox style home cage. Animals were recorded for at least an hour prior to drug administration to establish a stable temperature baseline. Following drug administration the recording was continued for 5 hours; thereafter animals were returned to the home cage in the vivarium. Drug challenges were conducted no more frequently than twice per week with a minimum three day interval between challenges. The order of doses was randomized across individuals for each test compound; ambient temperature conditions were alternated for each successive challenge day in the 4-methylmethcathinone experiment.

### Drugs

Drug doses were diluted in physiological saline and injected subcutaneously in a volume of 1 ml/kg. *d*-Methamphetamine was provided by RTI International (Research Triangle Park, NC) under contract from the National Institute on Drug Abuse (Bethesda, MD). 8-Hydroxy-*N*,*N*-dipropyl-2-aminotetralin (8-OH-DPAT) was purchased from Sigma-Aldrich (St. Louis, MO). The 4-methlymethcathinone used for this study was synthesized in gram quantities using a robust synthetic route based on literature precedent [31], with minor modifications, as follows.

#### α-Bromination

The preparation of 4-methylmethcathinone began from 4-methylpropiophenone (6.7 mmol) dissolved in glacial acetic acid (22 mL). To this solution, bromine (6.8 mmol) was added dropwise and the reaction stirred for two hours. After this time, the reaction was poured into a mixture of cold water and dichloromethane, and the organic layer separated. The organic layer was then repeatedly washed with aqueous sodium carbonate until the aqueous layer was basic on pH paper. The organic phase was then concentrated and the desired product isolated as colorless crystals after recrystallization from ether (1.4 g, quantitative).

#### 4-MMC

4′-Methyl-2-bromopropiophenone (22 mmol) was dissolved in dichloromethane (90 mL) and added dropwise to a solution of methylamine hydrochloride (1.5 g) and triethylamine (7 mL) in dichloromethane (200 mL). The reaction was then stirred at room temperature overnight, at which time aqueous HCl was added and the aqueous layer removed and washed with dichloromethane. The resulting aqueous solution was then made basic by addition of NaOH and the desired free amine extracted into dichloromethane. The solvent was then evaporated and resulting oil dissolved in anhydrous ether. HCl gas was then bubbled through the solution to generating a white solid, which was then recrystallized from isopropanol to give 4-MMC as a hydrochloride salt in 20% yield. The identity of the 4-MMC was confirmed by standard analytical methods (^1^H-NMR, ^13^C-NMR and MS) and the synthetic material is identical in all respects to that previously reported in the literature [31].

### Pharmacokinetic studies

Surgical placement of jugular vein catheters was conducted at the vendor prior to delivery at the laboratory. Blood samples were collected via the jugular catheters at timepoints 5, 15, 30, 60, 120 min and 3, 6 and 24 hrs after dosing. Plasma was stored at −80°C until analysis for drug concentrations via LC/MS/MS. Sample analysis was by LC/MS/MS with data analysis via the non compartmental analysis model of PK Solutions (Summit Research Services; Montrose, CO). The summary data for the Sprague-Dawley group have been previously described [32].

### Neurochemical studies

For microdialysis studies, drug naïve rats were implanted with a microdialysis guide cannula and probe using previously published procedures [33]. A guide cannula (SciPro, Sanborn, NY) was implanted aimed at the NAc shell (AP = +1.6 mm, bregma; ML = ±2.0 mm, bregma; DV = –5.0 mm, dura) [34], and rats were allowed one week for recovery. On the evening prior to the experiment, a 2 mm microdialysis probe was inserted into the guide cannula and perfused overnight with artificial CSF (0.1 µl/min) composed of the following (in mM): 149 NaCl, 2.8 KCl, 1.2 CaCl2, 1.2 MgCl2, 0.25 ascorbic acid, 5.4 D-glucose. The following morning, the perfusion rate was raised to 0.6 µl/min and samples were collected in 15 min fractions following 1 hr of equilibration.

### Data analysis

Analysis of the hourly averages of body temperature and summed activity data employed repeated measures analysis of variance (rmANOVA) with a between-subjects factor of rat strain, and within-subjects factors of drug treatment condition (dose) and time post-injection as outlined in the experiments. *Post-hoc* analyses of significant main effects for telemetry data were conducted using the Fisher's LSD test including all pairwise comparisons and for the neurochemical data using the Dunnett procedure; the criterion for significance was p<0.05 in all analyses. Analyses were conducted with GB-STATv7.0; Dynamic Microsystems, Silver Spring MD.

## Results

### 4-MMC

**Figure 1:**
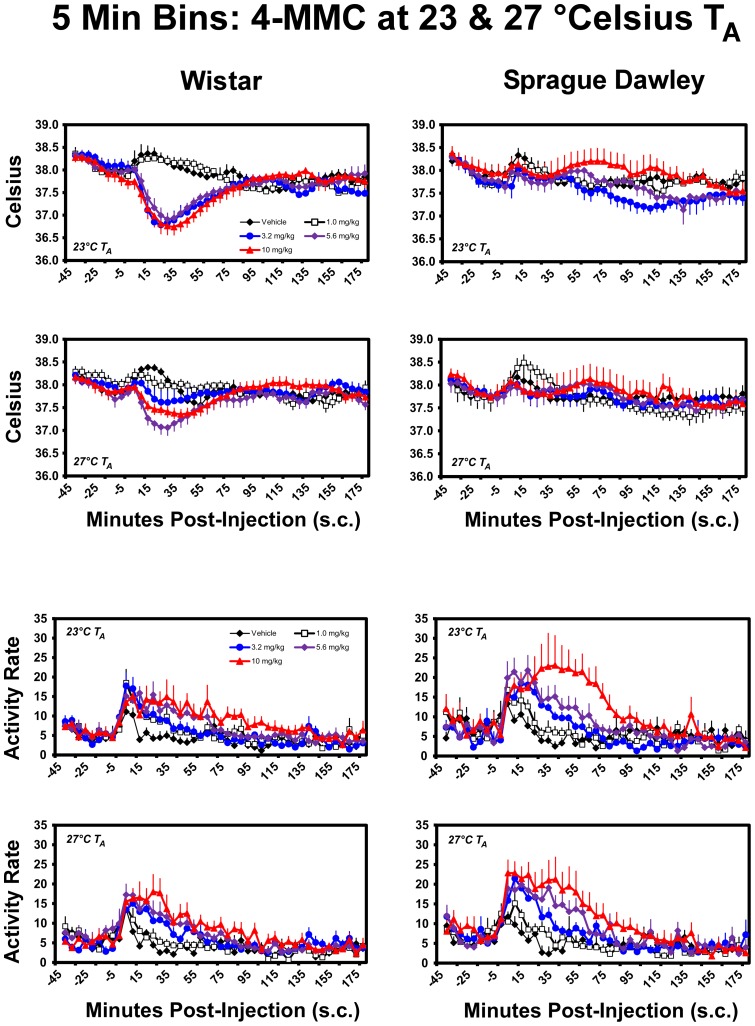
Mean (N = 8; ± SEM) body temperature and activity rates (count/min) are presented by the 5 minute sampling interval for Wistar and Sprague-Dawley rats following challenge with 4-MMC (1.0–10.0 mg/kg, s.c.) and vehicle under 23°C and 27°C T_A_ conditions.

**Figure 2:**
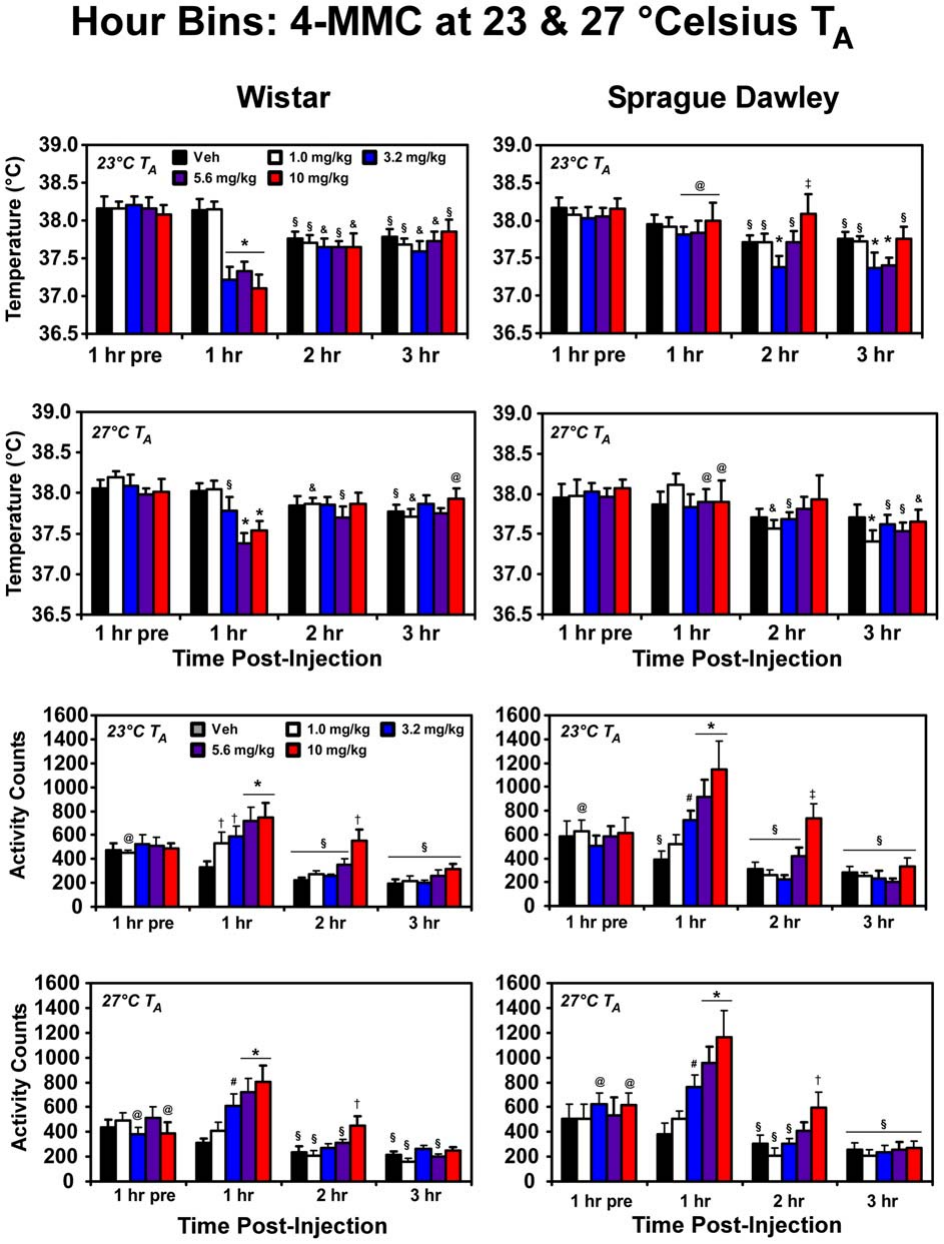
Statistical analysis was conducted on hourly bins (mean temperature and summed activity counts) for three hours after injection. *Post-hoc* confirmation of a significant difference from the following conditions is indicated by: Vehicle condition at a given timepoint: †; Pretreatment value within dose condition: §; Pretreatment baseline and vehicle condition at that time point: #; Vehicle condition, pretreatment value *and* between strains: *; Pretreatment value and between strains: &; Vehicle condition at the respective timepoint and between strains: ‡; Between strains: @.

Temperature was decreased in Wistar rats, but not Sprague-Dawley rats, when challenged with 4-MMC under both 23°C and 27°C ambient temperature conditions (Figure 1). A mean temperature nadir of 36.74 (±0.187)°C was observed following the administration of 10 mg/kg in the Wistar rats under 23°C ambient and 37.35 (±0.115)°C under 27°C ambient. One of the Wistar rats developed an infection in a minor wound and was euthanized; thus this individual is missing data for the vehicle at 23°C and the 3.2 mg/kg 4-MMC at 27°C conditions. The statistical analysis of the hourly averages (Figure 2) confirmed main effects of drug treatment (F_4,56_ = 4.48; p<0.005), time post-injection (F_3,42_ = 79.97; p<0.0001), as well as the interaction of strain with time post-injection (F_3,42_ = 17.07; p<0.0001), the interaction of dose condition with time post-injection (F_12,168_ = 5.76; p<0.0001) and the interaction of all three factors (F_12,168_ = 6.05; p<0.0001) under standard laboratory ambient temperature of 23°C. *Post-hoc* comparisons confirmed that body temperature in Wistar rats was significantly lowered in the first hour after injection of 3.2, 5.6 and 10 mg/kg 4-MMC compared to the pre-treatment baseline for each dose, compared with the first hour after saline and compared with the same doses in Sprague-Dawley rats. In contrast, the body temperature of Sprague-Dawley rats was lower than both the pretreatment baseline and the comparable timepoint after vehicle injection only following 3.2 mg/kg (2, 3 hrs) and 5.6 mg/kg (3 hrs) 4-MMC.

When administered under 27°C ambient temperature conditions, 4-MMC again altered body temperature as confirmed by a significant main effect of time post-injection (F_3,42_ = 18.95; p<0.0001), as well as the interaction of dose condition with time post-injection (F_12,168_ = 2.88; p<0.005). The analysis also confirmed a significant effect of the interaction of strain with time post-injection (F_3,42_ = 6.05; p<0.005). *Post-hoc* comparisons confirmed that body temperature was decreased in Wistar rats relative to their own pretreatment baseline, a comparable timepoint after saline administration and compared with Sprague-Dawley rats in the first hour after 5.6 or 10.0 mg/kg 4-MMC. The body temperature of Sprague-Dawley rats was unchanged in the first hour after 4-MMC administration, however the *post-hoc* test confirmed a reliable decrease in temperature in the third hour after the 1.0 mg/kg dose relative to the pretreatment baseline and the vehicle condition at the same timepoint.

Locomotor activity was increased by 4-MMC in both strains of rats when challenged under either ambient temperature condition (Figure 1). The statistical analysis confirmed main effects of drug treatment (F_4,56_ = 16.54; p<0.0001), time post-injection (F_3,42_ = 56.76; p<0.0001) and the interaction of dose condition with time post-injection (F_12,168_ = 8.84; p<0.0001) under standard laboratory ambient temperature of 23°C (Figure 2). Activity in the first hour after 5.6 or 10.0 mg/kg 4-MMC was significantly elevated over the hour prior to injection and the respective timepoint after saline administration in each strain of rats. The Sprague-Dawley activity was also increased for these doses/timepoints relative to the Wistar group. A significant increase in locomotor activity was likewise observed in the second hour after 10.0 mg/kg relative to the respective vehicle condition in each strain and the effect was greater in Sprague-Dawley rats.

Nearly identical effects on locomotor activity were observed at the 27°C ambient temperature. The statistical analysis confirmed main effects of drug treatment (F_4,56_ = 12.80; p<0.0001), time post-injection (F_3,42_ = 80.52; p<0.0001) and the interaction of dose condition with time post-injection (F_12,168_ = 9.28; p<0.0001). The *post-hoc* test confirmed that activity was higher in Wistar rats after the 3.2, 5.6 and 10.0 mg/kg doses of 4-MMC relative to the hour before injection and the comparative timepoint after vehicle injection. Activity was higher in the Sprague-Dawley group in the first hour after 5.6 or 10.0 mg/kg 4-MMC in comparison with the hour pre-injection, the same timepoint after vehicle injection and the same dose/time points in the Wistar rats.

### 4-MMC versus d-Methamphetamine

**Figure 3:**
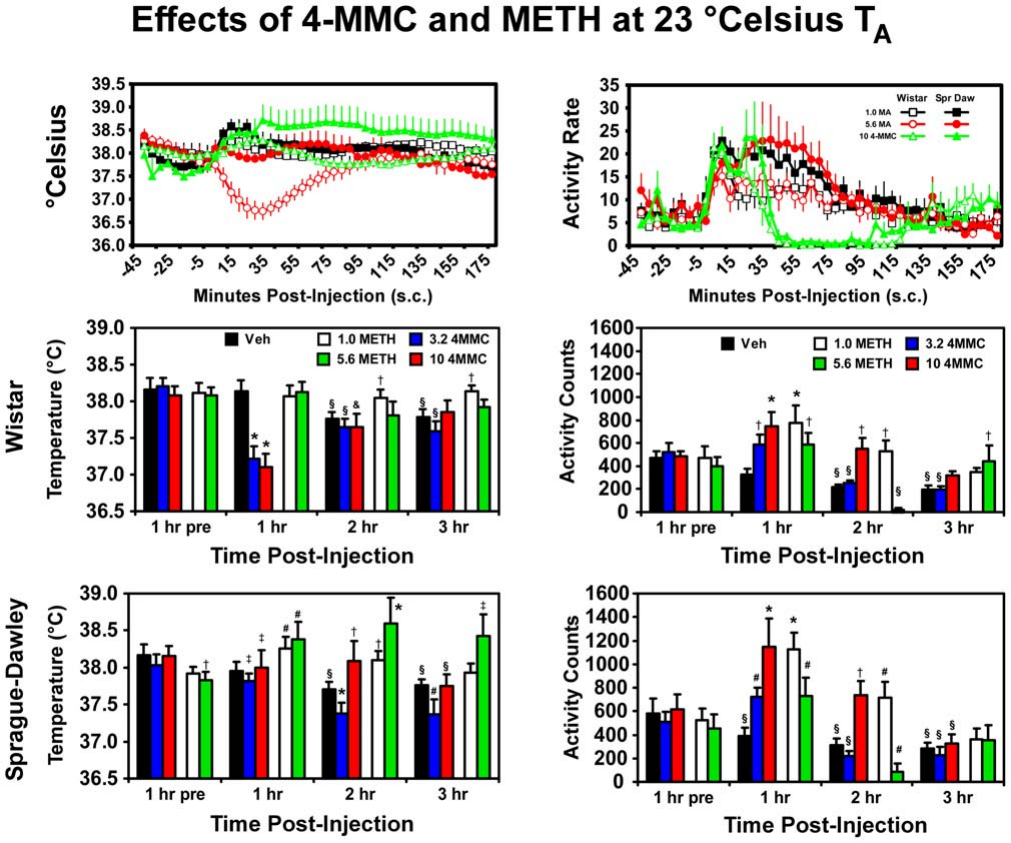
Mean (± SEM) body temperature and activity data are presented for Wistar (N = 7) and Sprague-Dawley (N = 8) rats following challenge with vehicle, d-methamphetamine (1.0, 5.6 mg/kg, s.c.) and 4-MMC (3.2, 10.0 mg/kg, s.c.) under 23°C T_A_. Data were collected at 5 minute intervals (upper panels) and were statistically analyzed as hourly averages. All other statistical conventions are as outlined in Figure 2.

Analysis was conducted to compare the effects of 1.0 or 5.6 mg/kg d-methamphetamine (METH) s.c. with the data collected for 0.0, 3.2 and 10.0 mg/kg challenge in the Wistar and Sprague-Dawley rats under 23°C ambient temperature conditions (i.e., the data illustrated in Figures 1 and 2). Body temperature was decreased by 4-MMC in the Wistar rats and increased by METH in the Sprague-Dawley rats as is illustrated in Figure 3. The statistical analysis of temperature data confirmed main effects of drug treatment (F_4,52_ = 10.33; p<0.0001), time post-injection (F_3,39_ = 13.37; p<0.0001), as well as the interaction of strain with time post-injection (F_3,39_ = 13.22; p<0.0001), the interaction of dose condition with time post-injection (F_12,156_ = 9.41; p<0.0001) and the interaction of all three factors (F_12,156_ = 5.80; p<0.0001). The *post-hoc* test confirmed that body temperature of Wistar rats was lower than pretreatment baseline, then after vehicle at the same timepoint and compared to Sprague-Dawley rats in the first hour after either 3.2 or 10.0 mg/kg 4-MMC. The temperature of Sprague-Dawley rats was decreased relative to both vehicle at the same timepoint and the pre-treatment baseline in the second and third hours after 3.2 mg/kg 4-MMC but increased relative to vehicle in the second hour after 10.0 mg/kg 4-MMC. Body temperature was also significantly increased in the Sprague-Dawley rats relative to the pretreatment baseline and vehicle condition in the first hour after 1.0 mg/kg METH and for the first two hours after 5.6 mg/kg METH; for this latter dose, temperature was elevated in the third hour relative to the vehicle condition. The temperature in the second and third hour after 5.6 mg/kg METH was reliably increased relative to the temperature of Wistar rats.

Locomotor activity was increased by 4-MMC and by METH in each strain of rats as is illustrated in Figure 3. The statistical analysis of activity data confirmed main effects of drug treatment (F_4,52_ = 15.45; p<0.0001), time post-injection (F_3,39_ = 46.10; p<0.0001), as well as the interaction of dose condition with time post-injection (F_12,156_ = 10.98; p<0.0001). *Post-hoc* analysis confirmed that locomotor activity was increased over pretreatment levels and vehicle in the first hour after 10.0 mg/kg 4-MMC or 1.0 mg/kg METH in both strains; greater activity was confirmed for the Sprague-Dawley rats relative to Wistar rats for both of these drug treatments. Activity was also reliably higher than pretreatment and the respective vehicle condition in the first hour after 3.2 mg/kg 4-MMC and 5.6 mg/kg METH in the Sprague-Dawley rats. Activity was confirmed higher than the vehicle condition in the first hour after 3.2 mg/kg 4-MMC or 5.6 mg/kg METH, in the second hour after 10.0 mg/kg 4-MMC and in the third hour after 5.6 mg/kg METH in the Wistar rats. In both strains locomotor activity was suppressed in the second hour after 5.6 mg/kg METH.

### 8-hydroxy-*N*,*N*-dipropyl-2-aminotetralin (8-OH-DPAT)

**Figure 4:**
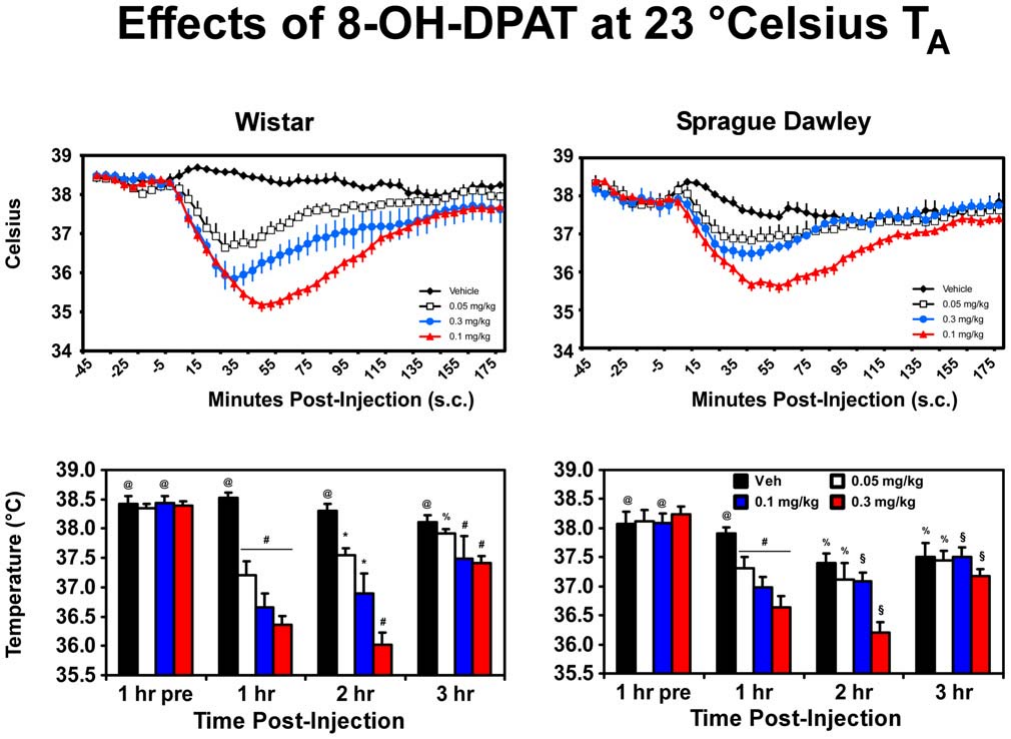
Mean (± SEM) body temperature is presented for Wistar (N = 7) and Sprague-Dawley (N = 7) rats following challenge with 8-OH-DPAT under 23°C T_A_ conditions. Data were collected at 5 minute intervals (upper panels) and are analyzed as hourly averages (lower panels). A reliable difference between strains (only) is indicated by %; all other statistical conventions are as outlined in Figure 2.

The serotonin receptor 5-HT_1A/7_ receptor agonist 8-OH-DPAT significantly reduced body temperature in each strain of rats (Figure 4). Following the 0.3 mg/kg dose, a mean temperature nadir of 35.18 (±0.178)°C was observed in the Wistar rats and a nadir of 35.62 (±0.177)°C was found in the Sprague-Dawley group. The statistical analysis confirmed main effects of drug treatment condition (F_3,36_ = 41.08; p<0.0001) and time post-injection (F_3,36_ = 128.34; p<0.0001) as well as an interaction of drug treatment with rat strain (F_3,36_ = 5.93; p<0.005), drug treatment and time post-injection (F_9,108_ = 25.12; p<0.0001) and the interaction of all three factors (F_9,108_ = 2.26; p<0.05).

*Post-hoc* comparisons of these effects confirmed that 8-OH-DPAT (0.05, 0.1, 0.3 mg/kg) lowered body temperature in the first hour relative to the vehicle condition and the pre-treatment baseline in Sprague-Dawley rats; this effect persisted to the second hour for the 0.3 mg/kg dose. The hypothermia relative to the vehicle condition and the pre-treatment baseline was also observed in Wistar rats for two hours after dosing with all three doses of 8-OH-DPAT and for the highest two doses body temperature remained below that of the vehicle condition. Body temperature of Wistar rats was significantly higher than that of Sprague-Dawley rats for all timepoints in the vehicle challenge condition, for the pre-treatment hour before the 0.1 mg/kg condition and for the second and third hours after 0.05 mg/kg 8-OH-DPAT.

### Pharmacokinetic studies

**Figure 5:**
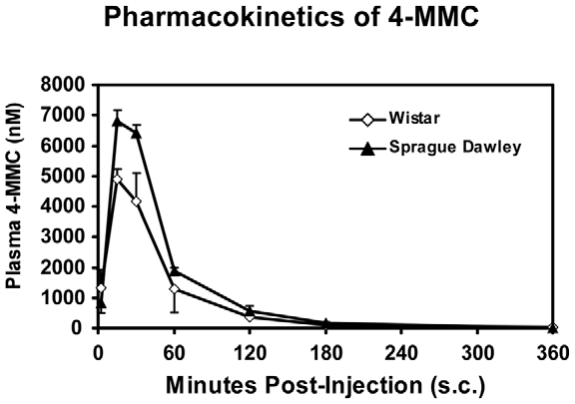
Mean (N = 3, ±SEM) plasma levels of 4-MMC up to 6 hrs after administration of 5.6 mg/kg 4-MMC, s.c.

The timecourse of elimination of 4-MMC from Sprague Dawley and Wistar rats was similar, as is illustrated in Figure 5. The peak concentrations (Cmax) observed following a 5.6 mg/kg subcutaneous dose were 1206 (Sprague Dawley) and 868 (Wistar) ng/mL of plasma (Table 1).

**Table 1:** Pharmacokinetic parameters for male Sprague Dawley (N = 3) and Wistar (N = 3) rats following subcutaneous administration of 5.6 mg/kg 4-MMC.

Strain	Tmax (hr)	Cmax (ng/mL)	Cmax (nM)	AUC∞ (area) ng-hr/mL
Sprague- Dawley	0.25	1206	6806	1170
Wistar	0.25	868	4901	870

### *In vivo* Microdialysis

**Figure 6:**
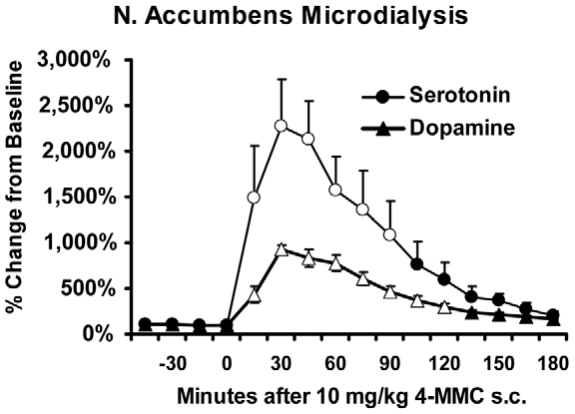
Serotonin and Dopamine levels in the nucleus accumbens (shell) region following 10 mg/kg 4-MMC, s.c. (N = 5, ±SEM). Open symbols indicate significant change from baseline.

A study in 5 male Wistar rats demonstrated that 10 mg/kg 4-MMC s.c. increased extra-cellular dopamine (DA; F(15,60) = 41.01, p<0.001) and serotonin (5-HT; F(15,60) = 10.80, p<0.001) in the nucleus accumbens shell (Figure 6). The Dunnett *post-hoc* test confirmed significant increases over the pre-treatment average for DA (2 hrs) and 5-HT (1.5 hrs). The baseline sample averages ranged from 2.20–2.31 nM for DA, 0.45–0.46 nM for 5-HT and the relative 4-MMC-induced *increase* was much greater for 5-HT (∼22-fold) than DA (∼9-fold). Probe placements were verified histologically at the conclusion of the study.

## Discussion

These experiments constitute a comprehensive investigation into the thermoregulatory and locomotor stimulant properties of 4-methylmethcathinone (4-MMC; aka mephedrone, MMCAT, “plant food”), a drug which is being used recreationally by humans [8], [9], [35]. The data show that 4-MMC has substantial effects on both body temperature and locomotor activity; it was found to lower body temperature in Wistar rats and act as a locomotor stimulant in both Wistar and Sprague-Dawley rats. It was found that 4-MMC is equally efficacious as d-methamphetamine (METH) in stimulating locomotion, however it was about tenfold less potent than METH.

The pronounced decrease in body temperature that was observed in the Wistar rats is reminiscent of the effects of 3,4-methylenedioxymethamphetamine [15], [16] and N-ethyl-3,4-methylenedioxyamphetamine [36], both of which reduce the body temperature of rats under normal laboratory ambient temperature conditions of ∼22–24°C. These observations contrast with prior studies showing that methcathinone [37] and cathinone [38] *increase* body temperature in male Sprague-Dawley and Wistar rats, respectively. There are data showing that khat chewing increases the body temperature of humans [39], however it is should be noted that MDMA consistently increases the body temperature of humans [28] and macaque monkeys [29] even under low ambient temperature conditions under which rodent temperature responses are in the negative direction. What was perhaps most surprising was that the body temperature response to 4-MMC was not qualitatively altered when administered across a range of ambient temperatures at which MDMA converts from a decrease to an increase in body temperature [16]. The present results therefore indicate that 4-MMC may have thermoregulatory actions that are in some ways distinct from those of MDMA.

The more modest effect of 4-MMC on the body temperature of Sprague-Dawley (vs. Wistar) rats is more likely attributable to the pharmacology of 4-MMC interacting with strain differences rather than to a relative resistance in Sprague-Dawley rats to exhibit hypothermia under normal laboratory ambient temperature conditions, since the 5-HT_1A_/5-HT_7_ receptor agonist 8-OH-DPAT decreased the body temperature of both strains. More specifically, these data also suggest that unlike MDMA, 4-MMC does not produce hypothermia through pharmacological activity at 5-HT_1A_ or 5-HT_7_ receptor subtypes [40]. Still, there may be strain-dependent effects that are specific to 4-MMC. For example a recent study reported that four sequential doses of 1.0 or 3.0 mg/kg 4-MMC s.c. administered two hours apart, and the intravenous self-administration of 4-MMC (mean 1.77–6.78 mg/kg) under ambient temperature of ≥27°C elevates the rectal temperature of Sprague-Dawley rats [10]. It is possible that rat strain, dose and schedule of administration, specific ambient temperature and restrained vs. unrestrained temperature measurement techniques contribute to observed differences.

In contrast to the thermoregulatory findings, the locomotor effects of 4-MMC were more straightforward in that 4-MMC consistently increased activity in the home cage setting. This is consistent with prior demonstrations that cathinone increases locomotor activity in rats [41], [42] and both cathinone and methcathinone act as locomotor stimulants in mice [43], [44]. The degree of locomotor stimulation following 10 mg/kg 4-MMC administration was approximately equivalent to that produced by 1 mg/kg d-methamphetamine. The fact that METH increased locomotor activity following 1.0 mg/kg but suppressed activity in the second hour after 5.6 mg/kg is consistent with prior results showing open field locomotor stimulation being replaced by stereotyped behavior at around 2–3 mg/kg [45]–[47]. The present data also show that 4-MMC increases locomotor activity in two rat strains, albeit to a slightly greater extent in the Sprague-Dawley rats. This strain difference contrasts with a prior report that 2 mg/kg amphetamine, s.c., induced a greater increase in locomotor activity in Wistar versus Sprague-Dawley rats [48].

The locomotor effects are consistent with a recently developing literature and differences are likely attributable to species, dose and locomotor assay. For example, one study that reported that 4-MMC produces less locomotor stimulation than amphetamine and more than MDMA [11] likely used a submaximal 4-MMC dose (see Figure 1) and a dose of amphetamine that approximates the peak of a typical inverted-U dose-response function [49], [50]. A recent finding in adolescent Wistar rats reported MDMA-typical [51] thigmotaxis, and locomotor stimulation greater than that produced by 2 mg/kg METH following high dose (15–30 mg/kg) 4-MMC [52]; it was not made clear that the dose of METH was at the peak of the inverted U function for animals of that strain and age. Work by Lopez-Arnau and colleagues reported that 5 mg/kg 4-MMC was more potent than 5 mg/kg MDMA in stimulating open field activity [53]. Here we have examined a broad dose –response function and found locomotor stimulant effects of 4-MMC that are present up to 10 mg/kg without induction of stereotypy, similar to the effects of 10 mg/kg MDMA [51], [54]–[56]. Our neurochemical data extend prior results [11], [23] to a higher dose and confirm that extracellular serotonin in the nucleus accumbens is increased to a greater extent than is dopamine after a 10 mg/kg 4-MMC, s.c., dose, coinciding with maximum locomotor stimulant effects in our telemetry studies. We have recently shown that running wheel activity is suppressed by 4-MMC or MDMA whereas METH produces an interverted U pattern of increased, then decreased, wheel activity [57]. This latter finding suggests wheel-based locomotor assays may offer improved discrimination between dopamine-dominant classical stimulants and the MDMA-like stimulants which produce significant serotonergic alterations.

The pharmacokinetic (PK) data are consistent with the observed time course of effects on temperature and activity. Maximum plasma levels of 4-MMC were observed within 15 minutes of subcutaneous dosing and that the drug was mostly eliminated within two hours; the temperature and activity effects lasted about 90 minutes. A strain difference was observed in the PK experiment with Sprague Dawley rats achieving significantly higher peak drug levels 15–30 minutes after dosing. Although it would be tempting to attribute the strain difference in locomotor stimulation to this PK difference, the observation that Wistar rats' body temperature was more affected advises caution. It may be the case that 4-MMC is converted to one or more active metabolites and a difference in this process might account for a different outcome on temperature and activity. Additional investigation will be needed to resolve the apparent difference in metabolism of 4-MMC in Wistar and Sprague Dawley rats.

In summary, this study provides the first experimental data on the thermoregulatory effects of 4-methylmethcathinone, a novel cathinone derivative being used recreationally under the name mephedrone. The data also showed that 4-MMC is a locomotor stimulant in rats with an efficacy similar to that of a peak locomotor stimulant dose (1 mg/kg) of methamphetamine, albeit with a several fold reduction in potency. Some caution is warranted, however, since 4-MMC and methamphetamine appear to be equally potent in improving visuo-spatial memory in rhesus monkeys, thus species differences may be critical [58]. The thermoregulatory impact of 4-MMC is to reduce body temperature (at least in Wistar rats). These effects are more similar to the 3,4-methylenedioxymethamphetamine (MDMA) amphetamine derivative and are likely related to indirect/direct serotonergic agonist properties; see [59] for review. It remains a question, however, as to why 4-MMC did not produce any elevation of body temperature at the higher ambient temperature condition as would be expected for MDMA. These behavioral pharmacological data are consistent with neurochemical and pharmacological evidence that 4-MMC acts to block or reverse the dopamine and serotonin transporters with potencies that are more similar to MDMA than to methamphetamine [10], [11], [23]; also see Figure 6. The clear strain differences in thermoregulatory effects are intriguing because they may indicate differential metabolism or selective differences in pharmacodynamic effects. Our pharmacokinetic data were similar, suggesting differences in pharmacodynamics may be the better target for future investigation. In conclusion, although some similarities to amphetamine class stimulants were observed and the locomotor effects were consistent with a limited prior literature on the effects of cathinone/methcathinone, clearly 4-MMC confers a unique constellation of behavioral and physiological properties in rodents. Further investigation is warranted to clearly delineate specific health risks of 4-MMC in the human recreational user.

## References

[1] Bluelight website. (RC's) Big mephedrone thread. Available: http://www.bluelight.ru/vb/showthread.php?t=400517. Accessed 2010 Sep 28.

[2] Google Trends website. Google Trends: mephedrone. Available: http://www.google.com/trends?q=mephedrone. Accessed 2011 Jan 11.

[3] DEA (2009) 4-Methylmethcathinone in Oregon. Microgram Bull 4(7): 61–62.

[4] Cuoco J (2010) Devil Tracks: the new drug popping up in Central Texas. KXXV News Channel 25 (ABC). Waco, TX.

[5] Michael J (2010) Substances producing ‘legal highs’ no longer legal. The Bismarck Tribune. Bismarck, ND.

[6] ONDCP, 2011. Statement from White House Drug Policy Director on Synthetic Stimulants, a.k.a “Bath Salts” [press release]. Available: http://ondcp.gov/news/press11/020111.html. Accessed 2011 Feb 3.

[7] DEA (2011) Request for Information on Synthetic Cathinones. Microgram Bulletin 44: 31–34.

[8] Iversen L, Adebowale V, Abdulrahim D, Arr-Jones G, Barnes M, et al. (2010) Consideration of the cathinones. Advisory Council on the Misuse of Drugs.Available http://www.homeoffice.gov.uk/publications/agencies-public-bodies/acmd1/acmd-cathinodes-report-2010?view=Binary. Accessed 2012 Apr 11.

[9] Sedefov R, Solberg U, Gallegos A, Almeida A (2010) Europol–EMCDDA Joint Report on a new psychoactive substance: 4-methylmethcathinone (mephedrone). Lisbon, Portugal: European Monitoring Centre for Drugs and Drug Addiction.

[10] Hadlock GC, Webb KM, McFadden LM, Chu PW, Ellis JD, et al (2011) 4-Methylmethcathinone (mephedrone): neuropharmacological effects of a designer stimulant of abuse. J Pharmacol Exp Ther. 2011 Nov 339(2): 530–6. 10.1124/jpet.111.184119 PMC3200001 21810934

[11] Kehr J, Ichinose F, Yoshitake S, Goiny M, Sievertsson T, et al (2011) Mephedrone, compared with MDMA (ecstasy) and amphetamine, rapidly increases both dopamine and 5-HT levels in nucleus accumbens of awake rats. British Journal of Pharmacology 164: 1949–1958. 21615721 10.1111/j.1476-5381.2011.01499.x PMC3246659

[12] Martinez-Clemente J, Escubedo E, Pubill D, Camarasa J (2011) Interaction of mephedrone with dopamine and serotonin targets in rats. Eur Neuropsychopharmacol. 2012 Mar 22(3): 231–6. 10.1016/j.euroneuro.2011.07.009 21824752

[13] Psychonaut Webmapping Research Group (2010) Mephedrone Report. London, UK: Institute of Psychiatry, King's College London. Available: http://www.psychonautproject.eu/documents/reports/Mephedrone.pdf. Accessed 2011 Sep 3.

[14] Winstock AR, Mitcheson LR, Deluca P, Davey Z, Corazza O, et al (2011) Mephedrone, new kid for the chop? Addiction (Abingdon, England) 106: 154–161. 10.1111/j.1360-0443.2010.03130.x 20735367

[15] Dafters RI (1994) Effect of ambient temperature on hyperthermia and hyperkinesis induced by 3,4-methylenedioxymethamphetamine (MDMA or “ecstasy”) in rats. Psychopharmacology (Berl) 114: 505–508. 7855209 10.1007/BF02249342

[16] Malberg JE, Seiden LS (1998) Small changes in ambient temperature cause large changes in 3,4-methylenedioxymethamphetamine (MDMA)-induced serotonin neurotoxicity and core body temperature in the rat. J Neurosci 18: 5086–5094. 9634574 10.1523/JNEUROSCI.18-13-05086.1998 PMC6792575

[17] Pedersen NP, Blessing WW (2001) Cutaneous vasoconstriction contributes to hyperthermia induced by 3,4-methylenedioxymethamphetamine (ecstasy) in conscious rabbits. J Neurosci 21: 8648–8654. 11606652 10.1523/JNEUROSCI.21-21-08648.2001 PMC6762811

[18] Blessing WW, Seaman B, Pedersen NP, Ootsuka Y (2003) Clozapine reverses hyperthermia and sympathetically mediated cutaneous vasoconstriction induced by 3,4-methylenedioxymethamphetamine (ecstasy) in rabbits and rats. J Neurosci 23: 6385–6391. 12867524 10.1523/JNEUROSCI.23-15-06385.2003 PMC6740548

[19] Geezaman DF (2009) Surprisingly Like E: experience with 4-Methylmethcathinone (ID 77952). Available: http://www.erowid.org/experiences/exp.php?ID=77952. Accessed 2010 Sep 29.

[20] Meph_Test (2009) Good Alternative to MDMA: experience with 4-Methylmethcathinone (Mephedrone) & Alcohol (ID 82321). Available: http://www.erowid.org/experiences/exp.php?ID=82321. Accessed 2010 Sep 29.

[21] Dal Cason TA, Young R, Glennon RA (1997) Cathinone: an investigation of several N-alkyl and methylenedioxy-substituted analogs. Pharmacol Biochem Behav 58: 1109–1116. 9408221 10.1016/s0091-3057(97)00323-7

[22] Johanson CE, Schuster CR (1981) A comparison of the behavioral effects of l- and dl-cathinone and d-amphetamine. J Pharmacol Exp Ther 219: 355–362. 7288625

[23] Baumann MH, Ayestas MA Jr, Partilla JS, Sink JR, Shulgin AT, et al (2012) The Designer Methcathinone Analogs, Mephedrone and Methylone, are Substrates for Monoamine Transporters in Brain Tissue. Neuropsychopharmacology 37: 1192–1203. 22169943 10.1038/npp.2011.304 PMC3306880

[24] Battaglia G, Brooks BP, Kulsakdinun C, De Souza EB (1988) Pharmacologic profile of MDMA (3,4-methylenedioxymethamphetamine) at various brain recognition sites. Eur J Pharmacol 149: 159–163. 2899513 10.1016/0014-2999(88)90056-8

[25] Baumann MH, Clark RD, Rothman RB (2008) Locomotor stimulation produced by 3,4-methylenedioxymethamphetamine (MDMA) is correlated with dialysate levels of serotonin and dopamine in rat brain. Pharmacol Biochem Behav 90: 208–217. 18403002 10.1016/j.pbb.2008.02.018 PMC2491560

[26] Green AR, Mechan AO, Elliott JM, O'Shea E, Colado MI (2003) The pharmacology and clinical pharmacology of 3,4-methylenedioxymethamphetamine (MDMA, “ecstasy”). Pharmacol Rev 55: 463–508. 12869661 10.1124/pr.55.3.3

[27] Myles BJ, Jarrett LA, Broom SL, Speaker HA, Sabol KE (2008) The effects of methamphetamine on core body temperature in the rat-part 1: chronic treatment and ambient temperature. Psychopharmacology (Berl) 198: 301–311. 18438646 10.1007/s00213-007-1061-z

[28] Freedman RR, Johanson CE, Tancer ME (2005) Thermoregulatory effects of 3,4-methylenedioxymethamphetamine (MDMA) in humans. Psychopharmacology (Berl) 183: 248–256. 16163516 10.1007/s00213-005-0149-6

[29] Von Huben SN, Lay CC, Crean RD, Davis SA, Katner SN, et al (2007) Impact of ambient temperature on hyperthermia induced by (+/−)3,4-methylenedioxymethamphetamine in rhesus macaques. Neuropsychopharmacology 32: 673–681. 16641942 10.1038/sj.npp.1301078 PMC2080863

[30] Clark JD, Baldwin RL, Bayne KA, Brown MJ, Gebhart GF, et al.. (1996) Guide for the Care and Use of Laboratory Animals. Washington D.C.: Institute of Laboratory Animal Resources, National Research Council. 125 p.

[31] Camilleri A, Johnston MR, Brennan M, Davis S, Caldicott DG (2010) Chemical analysis of four capsules containing the controlled substance analogues 4-methylmethcathinone, 2-fluoromethamphetamine, alpha-phthalimidopropiophenone and N-ethylcathinone. Forensic Sci Int 197: 59–66. 20074881 10.1016/j.forsciint.2009.12.048

[32] Miller ML, Creehan KM, Angrish D, Barlow DJ, Houseknecht KL, et al. (2012) Changes in ambient temperature differentially alter the thermoregulatory, cardiac and locomotor stimulant effects of 4-methylmethcathinone (mephedrone). Drug Alcohol Depend Jul 23 [Epub ahead of print] DOI: http://dx.doi.org/10.1016/j.drugalcdep.2012.07.003. 10.1016/j.drugalcdep.2012.07.003 PMC3491086 22832282

[33] Caille S, Alvarez-Jaimes L, Polis I, Stouffer DG, Parsons LH (2007) Specific alterations of extracellular endocannabinoid levels in the nucleus accumbens by ethanol, heroin, and cocaine self-administration. J Neurosci 27: 3695–3702. 17409233 10.1523/JNEUROSCI.4403-06.2007 PMC6672416

[34] Paxinos G, Watson C (1998) The Rat Brain in Stereotaxic Coordinates. 4^th^ edition. New York: Academic Press.

[35] Winstock AR, Marsden J, Mitcheson L (2010) What should be done about mephedrone? BMJ 340: c1605. 20332508 10.1136/bmj.c1605

[36] Bexis S, Docherty JR (2006) Effects of MDMA, MDA and MDEA on blood pressure, heart rate, locomotor activity and body temperature in the rat involve alpha-adrenoceptors. Br J Pharmacol. 10.1038/sj.bjp.0706688 PMC2189797 16491100

[37] Rockhold RW, Carlton FB Jr, Corkern R, Derouen L, Bennett JG, et al (1997) Methcathinone intoxication in the rat: abrogation by dextrorphan. Ann Emerg Med 29: 383–391. 9055779 10.1016/s0196-0644(97)70351-2

[38] Tariq M, Islam MW, al-Meshal IA, el-Feraly FS, Ageel AM (1989) Comparative study of cathinone and amphetamine on brown adipose thermogenesis. Life Sci 44: 951–955. 2927251 10.1016/0024-3205(89)90494-3

[39] Nencini P, Amiconi G, Befani O, Abdullahi MA, Anania MC (1984) Possible involvement of amine oxidase inhibition in the sympathetic activation induced by khat (Catha edulis) chewing in humans. J Ethnopharmacol 11: 79–86. 6147440 10.1016/0378-8741(84)90097-7

[40] Rusyniak DE, Zaretskaia MV, Zaretsky DV, DiMicco JA (2007) 3,4-Methylenedioxymethamphetamine- and 8-hydroxy-2-di-n-propylamino-tetralin-induced hypothermia: role and location of 5-hydroxytryptamine 1A receptors. J Pharmacol Exp Ther 323: 477–487. 17702902 10.1124/jpet.107.126169

[41] Banjaw MY, Fendt M, Schmidt WJ (2005) Clozapine attenuates the locomotor sensitisation and the prepulse inhibition deficit induced by a repeated oral administration of Catha edulis extract and cathinone in rats. Behav Brain Res 160: 365–373. 15863233 10.1016/j.bbr.2005.01.002

[42] Gordon TL, Meehan SM, Schechter MD (1993) Differential effects of nicotine but not cathinone on motor activity of P and NP rats. Pharmacol Biochem Behav 44: 657–659. 8451269 10.1016/0091-3057(93)90182-s

[43] Glennon RA, Yousif M, Naiman N, Kalix P (1987) Methcathinone: a new and potent amphetamine-like agent. Pharmacol Biochem Behav 26: 547–551. 3575369 10.1016/0091-3057(87)90164-x

[44] Zelger JL, Schorno HX, Carlini EA (1980) Behavioural effects of cathinone, an amine obtained from Catha edulis Forsk.: comparisons with amphetamine, norpseudoephedrine, apomorphine and nomifensine. Bull Narc 32: 67–81. 6911034

[45] Hughes RN, Greig AM (1976) Effects of caffeine, methamphetamine and methylphenidate on reactions to novelty and activity in rats. Neuropharmacology 15: 673–676. 1004691 10.1016/0028-3908(76)90035-6

[46] Itoh K, Fukumori R, Suzuki Y (1984) Effect of methamphetamine on the locomotor activity in the 6-OHDA dorsal hippocampus lesioned rat. Life Sci 34: 827–833. 6422178 10.1016/0024-3205(84)90199-1

[47] Kuczenski R, Segal DS, Cho AK, Melega W (1995) Hippocampus norepinephrine, caudate dopamine and serotonin, and behavioral responses to the stereoisomers of amphetamine and methamphetamine. J Neurosci 15: 1308–1317. 7869099 10.1523/JNEUROSCI.15-02-01308.1995 PMC6577819

[48] McDermott C, Kelly JP (2008) Comparison of the behavioural pharmacology of the Lister-Hooded with 2 commonly utilised albino rat strains. Prog Neuropsychopharmacol Biol Psychiatry 32: 1816–1823. 18727950 10.1016/j.pnpbp.2008.08.004

[49] Hall DA, Stanis JJ, Marquez Avila H, Gulley JM (2008) A comparison of amphetamine- and methamphetamine-induced locomotor activity in rats: evidence for qualitative differences in behavior. Psychopharmacology (Berl) 195: 469–478. 17874316 10.1007/s00213-007-0923-8 PMC2423722

[50] Mueller K, Kunko PM, Whiteside D, Haskett C (1989) Time course of amphetamine-induced locomotor stereotypy in an open field. Psychopharmacology (Berl) 99: 501–507. 2594917 10.1007/BF00589899

[51] Gold LH, Koob GF, Geyer MA (1988) Stimulant and hallucinogenic behavioral profiles of 3,4-methylenedioxymethamphetamine and N-ethyl-3,4-methylenedioxyamphetamine in rats. J Pharmacol Exp Ther 247: 547–555. 2903234

[52] Motbey CP, Hunt GE, Bowen MT, Artiss S, McGregor IS (2012) Mephedrone (4-methylmethcathinone, ‘meow’): acute behavioural effects and distribution of Fos expression in adolescent rats. Addict Biol 17: 409–422. 21995495 10.1111/j.1369-1600.2011.00384.x

[53] Lopez-Arnau R, Martinez-Clemente J, Pubill D, Escubedo E, Camarasa J (2012) Comparative neuropharmacology of three psychostimulant cathinone derivatives: butylone, mephedrone and methylone. Br J Pharmacol. Apr 18 [Epub ahead of print] DOI: http://dx.doi.org/10.1111/j.1476-5381.2012.01998.x. 10.1111/j.1476-5381.2012.01998.x PMC3481047 22509960

[54] Bankson MG, Cunningham KA (2002) Pharmacological studies of the acute effects of (+)-3,4-methylenedioxymethamphetamine on locomotor activity: role of 5-HT(1B/1D) and 5-HT(2) receptors. Neuropsychopharmacology 26: 40–52. 11751031 10.1016/S0893-133X(01)00345-1

[55] Walker QD, Williams CN, Jotwani RP, Waller ST, Francis R, et al (2007) Sex differences in the neurochemical and functional effects of MDMA in Sprague-Dawley rats. Psychopharmacology (Berl) 189: 435–445. 17019566 10.1007/s00213-006-0531-z

[56] Gilpin NW, Wright MJ Jr, Dickinson G, Vandewater SA, Price JU, et al (2011) Influences of activity wheel access on the body temperature response to MDMA and methamphetamine. Pharmacol Biochem Behav 99: 295–300. 21605589 10.1016/j.pbb.2011.05.006 PMC3129476

[57] Huang PK, Aarde SM, Angrish D, Houseknecht KL, Dickerson TJ, et al. (2012) Contrasting effects of d-methamphetamine, 3,4-methylenedioxymethamphetamine, 3,4-methylenedioxypyrovalerone, and 4-methylmethcathinone on wheel activity in rats. Drug Alcohol Depend. Jun 2 [Epub ahead of print] DOI: http://dx.doi.org/10.1016/j.drugalcdep.2012.05.011. 10.1016/j.drugalcdep.2012.05.011 PMC3439532 22664136

[58] Wright MJ Jr, Vandewater SA, Angrish D, Dickerson TJ, Taffe MA (2012) Mephedrone (4-methylmethcathinone, 4MMC) and d-methamphetamine improve visuo-spatial associative memory, but not spatial working memory, in rhesus macaques. Br J Pharmacol 2012 Jul 2. Epub ahead of print. doi: h10.1111/j.1476-5381.2012.02091.x. 10.1111/j.1476-5381.2012.02091.x PMC3504998 22748013

[59] Docherty JR, Green AR (2010) The role of monoamines in the changes in body temperature induced by 3,4-methylenedioxymethamphetamine (MDMA, ecstasy) and its derivatives. British Journal of Pharmacology 160: 1029–1044. 20590597 10.1111/j.1476-5381.2010.00722.x PMC2936013

